# Peroxidases Bound to the Growing Lignin Polymer Produce Natural Like Extracellular Lignin in a Cell Culture of Norway Spruce

**DOI:** 10.3389/fpls.2016.01523

**Published:** 2016-10-18

**Authors:** Tino Warinowski, Sanna Koutaniemi, Anna Kärkönen, Ilari Sundberg, Merja Toikka, Liisa Kaarina Simola, Ilkka Kilpeläinen, Teemu H. Teeri

**Affiliations:** ^1^Department of Agricultural Sciences, Viikki Plant Science Centre, University of HelsinkiFinland; ^2^Department of Food and Environmental Chemistry, University of HelsinkiFinland; ^3^Laboratory of Organic Chemistry, Department of Chemistry, University of HelsinkiFinland; ^4^Department of Biosciences, University of HelsinkiFinland

**Keywords:** dehydrogenation polymer, lignin-carbohydrate complex, lignin polymerization, oxidative enzymes, peroxidase, polymer oxidation

## Abstract

Lignin, an important component of plant cell walls, is a polymer of monolignols derived from the phenylpropanoid pathway. Monolignols are oxidized in the cell wall by oxidative enzymes (peroxidases and/or laccases) to radicals, which then couple with the growing lignin polymer. We have investigated the characteristics of the polymerization reaction by producing lignin polymers *in vitro* using different oxidative enzymes and analyzing the structures formed with NMR. The ability of the enzymes to oxidize high-molecular-weight compounds was tested using cytochrome *c* as a substrate. The results support an idea that lignin structure is largely determined by the concentration ratios of the monolignol (coniferyl alcohol) and polymer radicals involved in the coupling reaction. High rate of the lignin polymer oxidation compared to monolignol oxidation leads to a natural-like structure. The high relative rate can be achieved by an open active site of the oxidative enzyme, close proximity of the enzyme with the polymeric substrate or simply by high enzymatic activity that consumes monolignols rapidly. Monolignols, which are oxidized efficiently, can be seen as competitive inhibitors of polymer oxidation. Our results indicate that, at least in a Norway spruce (*Picea abies* L. Karst.) cell culture, a group of apoplastic, polymer-oxidizing peroxidases bind to the lignin polymer and are responsible for production of natural-like lignin in cell suspension cultures *in vivo*, and also *in vitro*. The peroxidases bound to the extracellular lignin had the highest ability to bind to various cell wall polymers *in vitro*. Extracellular lignin contains pectin-type sugars, making them possible attachment points for these cationic peroxidases.

## Introduction

Lignin is a complex, hydrophobic component in the plant cell wall of water-conducting vessels and tracheids, as well as support-giving sclerenchyma cells. It gives cell walls resilience against both mechanical stress and biological perils, and contributes to the calorific value of wood as a fuel. However, for pulp and paper manufacture as well as in biofuel production lignin is an obstruction that needs to be removed or broken down in processes that are both economically and environmentally challenging. Some lignin structures are more susceptible to degradation than others. Achieving control of lignin content as well as subunit and linkage composition would thus create significant benefits (Grabber, 2005).

**Figure 1:**
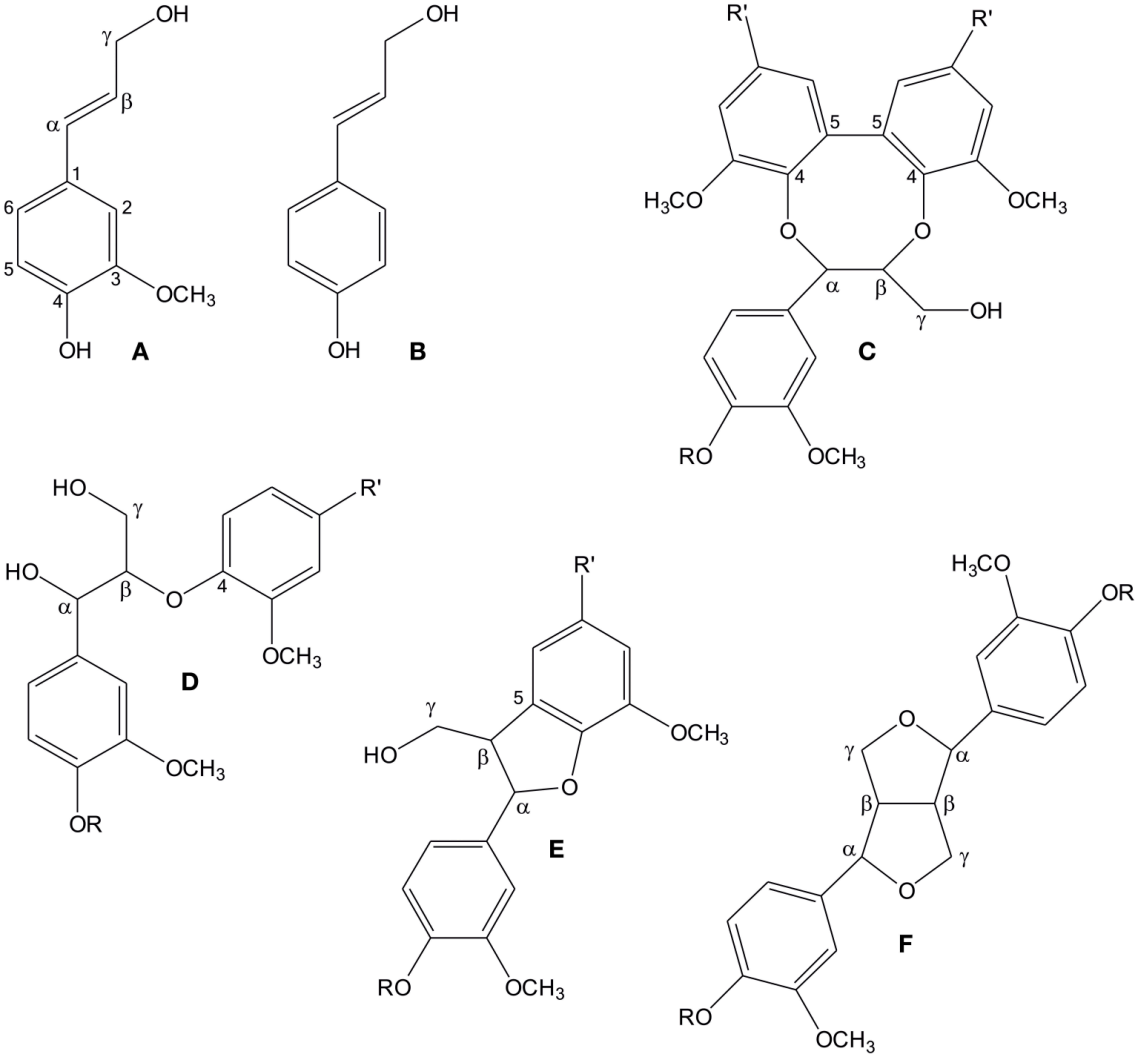
**The structures of spruce lignin monomers coniferyl alcohol (A) and ***p***-coumaryl alcohol (B), as well as four principal substructures in softwood lignin: 5-5-***O***-4 (C), β-***O***-4 (D), β-5 (E), and β-β (F)**. R denotes a continuing lignin molecule, or a hydrogen atom, if the molecule ends at that point. Similarly, R' denotes either a continuing lignin molecule or a phenylpropanoid side chain.

Lignin is a polymer of monolignols, which are derivatives of the phenylpropanoid pathway. The principal monolignol in gymnosperms is coniferyl alcohol, supplemented with a small quantity of *p*-coumaryl alcohol (Figure 1; Boerjan et al., 2003). Monolignols are oxidized in the apoplast into resonance-stabilized radicals by the action of oxidative enzymes, which can be either peroxidases (class III; EC 1.11.1.7; donor:hydrogen peroxide oxidoreductase) or laccases (*p*-diphenol:oxygen oxidoreductase, EC 1.10.3.2). The radicals then couple *in muro* to form the lignin polymer (reviewed by Vanholme et al., 2010; Wang et al., 2013). Both peroxidases and laccases are present in the apoplast as numerous isoenzymes with wide substrate specificities (Koutaniemi et al., 2005, 2015; Berthet et al., 2011; Turlapati et al., 2011; Novo-Uzal et al., 2013; Barros et al., 2015). It is now known that both laccases and peroxidases contribute to lignin biosynthesis at least in Arabidopsis (*Arabidopsis thaliana*; Berthet et al., 2011; Novo-Uzal et al., 2013; Zhao et al., 2013; Shigeto and Tsutsumi, 2016).

Monolignol radicals can couple in various ways to create different substructures in lignin, which, in principle, leads to a random structure in lignin. The four main substructures are β-*O*-4, β-5, β-β and 5-5-*O*-4 (dibenzodioxocin) linkages (Figure 1; Adler, 1977; Karhunen et al., 1995). However, it has been shown that the relative abundances of these substructures vary in different parts of the cell wall. For example, in Norway spruce (*Picea abies* (L.) Karst.), the 5-5-*O*-4 structure is enriched in the innermost part of the tracheid secondary cell wall (Kukkola et al., 2003). The parameters which determine the abundance of lignin substructures have been under active evaluation. The classical (combinatorial) model of lignin polymerization states that the structure of the polymer is determined by the chemistry of the coupling reaction, where proteins play no role beyond production of phenolic radicals. Polymerization would thus be a stochastic process (Ralph et al., 2004). The alternative hypothesis about dirigent protein-mediated coupling of monolignols in lignin formation (Gang et al., 1999; Davin and Lewis, 2005) has not been supported by scientific data, although a recent paper suggests that dirigent proteins have a role in lignin formation in the Casparian strip of root endodermis (Hosmani et al., 2013). Factors already shown to affect the types of bonds created include concentration of monolignols (Adler, 1977; Terashima et al., 1995; Syrjänen and Brunow, 2000) and pH (Ämmälahti and Brunow, 2000). Moreover, the carbohydrate matrix of the cell wall has been suggested to influence the structure of lignin, as spherical lignin particles were detected in the middle lamella within the random pectin network, whereas lamellar lignin particles were detected in the secondary cell wall that contains uniformly oriented cellulose microfibrils (Donaldson, 1994; Wi et al., 2005).

Formation of a covalent bond between a monolignol and the growing lignin polymer requires the presence of an unpaired electron in both entities, meaning that also the polymer must be oxidized during the process. In natural lignin, growth of the polymer takes place in an end-wise manner, i.e., monolignols are sequentially added to the polymer (Brunow et al., 1998). Oxidation of the polymer may happen by a radical transfer between the monolignol radical and the polymer, but this mechanism has been shown to be inefficient (Sasaki et al., 2004). Also, a shuttle mechanism over the plasma membrane involving, for example, manganese ions that would oxidase monolignols/lignin polymer in the apoplast has been suggested (Ōnnerud et al., 2002). Alternatively, the enzymes may oxidize lignin directly. It has been shown that poplar (*Populus alba*) has a cell wall-bound peroxidase capable of directly oxidizing high-molecular-weight lignin polymers (Sasaki et al., 2004). *In vitro* this enzyme produces from monolignols and hydrogen peroxide a dehydrogenation polymer (DHP), which has structural similarities with natural lignins. The ability of peroxidases to oxidize lignin directly was concluded to be important in the production of natural lignin polymers (Sasaki et al., 2004). As such, the 5-5-*O*-4 substructures are indicative of lignin polymer oxidation, since these substructures are not found in lignin oligomers and are found at reduced amounts in *in vitro*-produced DHPs (Ralph, 1999).

In this study, we have used a Norway spruce tissue culture as a model system, as the cells produce extracellular lignin in the culture medium (Simola et al., 1992; Kärkönen et al., 2002; Koutaniemi et al., 2007; Kärkönen and Koutaniemi, 2010). Structurally, this polymer resembles natural lignins (Brunow et al., 1990, 1993, 1998; Koutaniemi et al., 2005). A high number of peroxidase isoenzymes and one laccase are present in the culture medium (Kärkönen et al., 2002; Koutaniemi et al., 2015), resembling in this sense the developing xylem of Norway spruce (Fagerstedt et al., 2010). Interestingly, a specific subset of cationic peroxidases and another laccase isoenzyme are bound to the extracellular lignin (Koutaniemi, 2007; Koutaniemi et al., 2015). Purified culture medium laccase and peroxidases have a high affinity to coniferyl alcohol and are able to form high-molecular-weight DHP from coniferyl alcohol *in vitro* (Kärkönen et al., 2002; Koutaniemi et al., 2005, 2015). In the present work, we have used oxidative enzymes isolated from the culture medium and from the extracellular lignin to produce DHPs for structural studies. Fungal laccases from lignin-degrading fungi *Trametes hirsuta* and *Melanocarpus albomyces* were also utilized as these enzymes are known to be able to oxidize polymeric lignin (Kiiskinen et al., 2002; Rittstieg et al., 2002). As pectin-type sugars were detected to be bound to extracellular lignin, we assessed binding of the enzymes extracted from the extracellular lignin and other sources to cell wall components and polymers resembling them. We suggest that the extracellular lignin-bound fraction of the secreted suspension culture peroxidases is specialized in lignin polymerization and, more generally, that the concentration ratio of polymer and monomer radicals is a key factor that determines lignin structure.

## Materials and methods

### Tissue culturing and enzyme harvesting

The Norway spruce tissue culture line A3/85, which forms extracellular lignin in the culture medium (Simola et al., 1992), was maintained and propagated on a solid nutrient medium and transferred to liquid medium for lignin production (Kärkönen et al., 2002; Koutaniemi et al., 2005). Extracellular lignin was collected by centrifugation and the soluble enzymes from the culture medium were harvested by concentrating the medium by ultrafiltration.

Prior to protein extraction, extracellular lignin was washed several times with water until no peroxidase activity was released. The polymer-bound proteins were extracted twice (1 h each, at +4°C, with slow mixing) with 20 mM MES-buffer, pH 6.5 supplemented with 1 M NaCl, 1 mM CaCl_2_, and 1 mM phenylmethylsulfonyl fluoride (PMSF). Proteins were concentrated by ultrafiltration (Pall Stirred Cell Omega 10K system) and excess salt was removed using a PD-10 desalting column (GE Healthcare) equilibrated with the same buffer except that NaCl concentration was 50 mM. The extracted proteins were separated in an isoelectric focusing gel and stained for peroxidase activity with 0.83 mM 3,3′-diaminobenzidine in 50 mM Na-citrate buffer, pH 5.0 containing 10 mM CaCl_2_ and 0.5 mM H_2_O_2_ (Kärkönen et al., 2002).

### Purification of extracellular lignin-bound peroxidases and isolating the corresponding cDNA molecules

During protein purification, peroxidase activity in the fractions was screened on a microplate format using 0.67 mM ABTS (2,2-azino-bis(3-ethylbenzothiazoline-6-sulfonic acid), Sigma-Aldrich) and 0.4 mM H_2_O_2_ in 50 mM Na-acetate buffer, pH 5.0, with detection at 412 nm in a Multiscan MCC/340 plate reader (Labsystems). The peroxidase fraction was partially purified essentially as described in Koutaniemi et al. (2005). Briefly, the extracted protein fraction was brought to 2 M ammonium sulfate concentration and centrifuged at 10,000 × g for 20 min. The soluble fraction was loaded into a Phenyl Sepharose 6 FF column (GE Healthcare) equilibrated with 20 mM MES pH 6.0, 2 M ammonium sulfate and 1 mM CaCl_2_. Proteins were eluted with a step gradient to the same buffer without ammonium sulfate. Fractions containing peroxidase activity were loaded onto a Concanavalin A Sepharose column (GE Healthcare) equilibrated with 20 mM MES pH 6.0, 1 M ammonium sulfate, 1 mM CaCl_2_, MnCl_2_ and MgCl_2_ each and eluted with 500 mM methyl α-D-mannopyranoside (Fluka) in the same buffer. Eluted proteins were concentrated with a Omega 10K Stirred Cell System (Pall Life Sciences) and loaded into a Superdex 75 size exclusion chromatography column equilibrated with 20 mM MES pH 6.0, 50 mM NaCl, 1 mM CaCl_2_. Peroxidases from the size exclusion step were loaded into a SP Sepharose High Performance cation exchange column (GE Healthcare) equilibrated with 20 mM MES pH 6.0, 50 mM NaCl, 1 mM CaCl_2_. Basic peroxidases were eluted with a linear gradient to 300 mM NaCl in the same buffer and concentrated as above.

The pool of basic peroxidases was separated in a 10% SDS-PAGE gel. Proteins in the 32–42 kDa range were excised from the gel, subjected to in-gel digestion with trypsin and alkylated. The tryptic fragments were sequenced with LC-MS/MS tandem mass spectrometry as described in Poutanen et al. (2001).

Degenerate primers (5′-GGWGTC GTGTCRTGTGCWGACATTCTC-3′, 5′-CTCATCTCAMTITTTCARGCTCA-3′, 5′-CTGGTRCTGTAGGAYTCAAC-3′) were designed based on obtained peptide sequences (CADIL, LISLFQAQ, VESYSTSTHAFFK) showing similarity to peroxidases. Total and mRNA were isolated from the tissue-cultured spruce cells and reverse transcribed to cDNA as described in Koutaniemi et al. (2005). Partial peroxidase cDNA sequences were amplified with a polymerase chain reaction using the degenerate primers. Rapid amplification of cDNA ends (3′ RACE) was done using oligo-dT as the 3′ primer. Full-length 5′ RACEs were performed with SMART RACE cDNA Amplification Kit (BD Biosciences Clontech). Gene specific reverse primers were designed based on the 5′ untranslated regions of the genes and used in 3′ RACE (BD Biosciences Clontech) to obtain the full-length sequences. Three full-length cDNAs, each coding for a few sequenced peptides, were recovered. Primers used for cloning are shown in Supplementary Table 1.

### Production of DHP

Two different enzyme samples from the Norway spruce tissue culture were used in the DHP synthesis, total soluble proteins from the culture medium and extracted lignin-bound proteins (Table 1). In addition, DHP was produced with horseradish peroxidase (HRP) and lignin-degrading fungal laccases from *T. hirsuta* and *M. albomyces* (Table 1). Both hydrogen peroxide (34 mM, 5 ml) and coniferyl alcohol (Fluka, 34 mM, 5 ml) were simultaneously injected at a constant rate (250 μl h^−1^) using a micro pump (Harvard Apparatus) during a 20 h-period into 5 ml of enzyme solution containing a certain amount of coniferyl alcohol-oxidizing activity (**Table 3** for activity amounts) in a stirred flask at room temperature. After injection, the reaction was allowed to proceed for additional 4 h. With laccases, the hydrogen peroxide was omitted and replaced with the buffer. Polymers were collected by centrifugation (2500 g, 15 min), washed three times with water and lyophilized. All reactions were performed in 50 mM sodium acetate, pH 5.0, supplemented with 2.5 mM CaCl_2_. pH 5.0 was chosen to approximate the pH of the spruce cell cultures during extracellular lignin formation (pH 4.2–4.8; Kärkönen et al., 2002) and, on the other hand, the pH optimum of the isolated and total culture medium peroxidases (pH 4.0–6.5; Koutaniemi et al., 2005, and unpublished data). As pH affects the structure of DHPs produced (Ämmälahti and Brunow, 2000), DHP production by HRP was also conducted at pH 5.0.

**Table 1:** **Peroxidase and laccase samples used in this study**.

**Abbreviation**	**Description**	**Source/references**
Pa-SL	*Picea abies* total soluble culture medium proteins	Kärkönen et al., 2002
Pa-BD	*Picea abies* total proteins that bind the extracellular lignin	Koutaniemi, 2007; this study
HRP	Horseradish peroxidase type II	Sigma-Aldrich
MP-11	Microperoxidase	Sigma-Aldrich
Mal LAC	*Melanocarpus albomyces* laccase	Kiiskinen et al., 2002
Thi LAC	*Trametes hirsuta* laccase	Rittstieg et al., 2002

### Structural analysis of DHPs and lignin

Milled wood lignin (MWL) was prepared according to Björkman (1956) with some modifications. Sawdust was first extracted in a Soxhlet apparatus with acetone (48 h) and acetone-water 9:1 (4 h), then dried under vacuum. Dry sawdust was milled in a ball mill (Fritsch Pulverisette 6) at 320 rpm for 50 h and the wood powder was extracted with dioxane-water 9:1 (2 h) in a cooled ultrasonic bath. Dioxane-water was evaporated with a rotavapor nearly to the dryness, glacial acetic acid was added and the mixture was stirred at +50°C for 2 h. The filtered acetic acid solution was poured into water whereupon lignin precipitated. The precipitated lignin was collected by centrifugation, washed 1–2 times with water and dried.

For NMR analyses MWL, isolated extracellular lignin and DHPs were acetylated. In a typical procedure, the lignin sample (5–50 mg) was suspended into 10 ml of acetanhydride/pyridine (1:1, v/v). After stirring for 12 h at room temperature under argon, an excess of ethanol was added and the solvent was evaporated in a rotavapor. Successive ethanol addition and evaporation steps were performed until all residual pyridine, acetic acid and ethyl acetate were removed from the sample. The dry, acetylated lignin was dissolved into 0.7 ml of CDCl_3_ or deuterated DMSO (Cambridge Isotope Laboratories). The NMR spectra were measured with Varian Unity Inova 600 spectrometer (^1^H frequency 600 MHz). A modified version of Q-HSQC (Heikkinen et al., 2003), QQ-HSQC (Koskela et al., 2005) was applied to obtain quantitative 2D NMR spectra. The spectral width was 6000 Hz in ^1^H and 15,000 Hz in ^13^C. The number of scans was 256 (1024 data points) and the number of time increments 128 (hypercomplex method) with a repetition rate of 3 s, yielding measurement time of ~52 h for each spectrum. The spectra were zero-filled to 2048*1024 data points and apodized with squared bell window functions in both dimensions prior to Fourier transformation. All spectra were processed and analyzed with the standard VNMR software (Agilent Technologies). The relative amounts of the main structural units of lignin samples were calculated as percentage values from the sum of integral values of β-*O*-4, β-5, β-β and 5-5-*O*-4 substructures (Heikkinen et al., 2003). In order to give equal weight to the different bond types that occur in the polymer, standard scores were calculated. For each bond type, the standard score **z** has average value of 0 and variance of 1 across the samples.

### Enzyme activity measurements

Coniferyl alcohol peroxidase was determined at 262 nm with 100 μM coniferyl alcohol (Fluka) and 250 μM H_2_O_2_ in 45 mM Na-citrate buffer, pH 5.0 (Kärkönen et al., 2002). Coniferyl alcohol oxidase activity was measured at 262 nm using 70 μM coniferyl alcohol in 45 mM Na-acetate buffer, pH 4.5, supplemented with 125 U ml^−1^ catalase (Sigma C-100). Measurements were done in triplicate, and ε of 14,000 M^−1^ cm^−1^ for coniferyl alcohol was used for quantitative calculations (Kärkönen et al., 2002).

The ability of peroxidases to oxidize a high-molecular-weight substrate, cytochrome *c* from bovine heart (Sigma-Aldrich), was measured by following a decrease in the absorbance of the reduced ferrocytochrome *c* at 550 nm (ε = 18,500 M^−1^ cm^−1^; Hodges and Leonard, 1974; Sasaki et al., 2004). First, cytochrome *c* was reduced with 10 mM Na-dithionite for 30 min. Dithionite was removed by passing the sample through a PD-10 column (GE Healthcare) equilibrated with degassed 50 mM Na-citrate buffer, pH 5.0. Oxidation of cytochrome *c* was measured in the same buffer at room temperature using 40 μM cytochrome *c* and 1.2–4.6 nkat coniferyl alcohol peroxidase activity (soluble culture medium and lignin-bound proteins) or 10 μg HRP (~5500 nkat coniferyl alcohol peroxidase activity). Non-enzymatic oxidation was first followed for a few minutes, and peroxidase action was started with addition of 50 μM H_2_O_2_. The rate of non-enzymatic oxidation was subtracted from the peroxidase-catalyzed reaction. Results were normalized by calculating the ratio of cytochrome *c* peroxidase activity to coniferyl alcohol peroxidase activity (CytCprx/CAprx). Microperoxidase (MP-11, Sigma-Aldrich), which contains a heme of cytochrome *c* molecule with amino acids 11–21 still attached, was used as a positive control (34 nkat coniferyl alcohol peroxidase activity / reaction), as it represents an enzyme with a wide-open active site capable of accommodating high-molecular-weight substrates.

The ability of the *M. albomyces* and *T. hirsuta* laccases to oxidize DHP produced with HRP (see below) was determined by measuring changes in oxygen concentration with the Oxygraph oxygen electrode (Hansatech Instruments). The measurements were done in an air-saturated 50 mM Na-acetate buffer, pH 5.0, supplemented with 2.5 mM CaCl_2_, to which a fixed amount of laccase (1.2 nkat coniferyl alcohol oxidase activity) and 1 mg of suspended DHP were added (total volume 1 ml). The measuring cell was kept under magnetic stirring to prevent sedimentation of the polymer.

### Enzyme binding assays

Binding assays were performed to study the ability of peroxidases and laccases to bind to lignin and to lignin-like polymers (MWL, extracellular lignin, deglycosylated extracellular lignin and DHP produced with HRP), or to anionic polysaccharides (polygalacturonic acid and alginate). The tested enzyme samples included total soluble proteins from the culture medium, extracted lignin-bound proteins and HRP, as well as fungal laccases from *M. albomyces* and *T. hirsuta*. Control reactions contained the equal amount of enzymes without any polymer, and were treated similarly to the samples. Binding assays were conducted at pH 4.5 to approximate the pH of the culture medium during the formation of extracellular lignin.

The enzyme sample (2.5 nkat of coniferyl alcohol (per)oxidase activity) was diluted with the binding buffer (50 mM sodium tartrate, pH 4.5, 50 mM NaCl, 4 mM CaCl_2_, 1 mM PMSF, 0.01% (w/v) BSA) to a volume of 0.8 ml. Polymer (15–50 mg depending on availability; however, as polysaccharides form a large gel-volume when mixed with Ca^2+^ ions, a lower amount of polysaccharide polymers was used) was added and the sample was incubated for 2 h at +6°C with a slow mixing. After centrifugation (21,000 g, 4 min) the pellet was washed five times with the binding buffer, and the polymer-bound enzymes were eluted by extracting twice with 0.7 ml of the extraction buffer (20 mM MES, pH 6.0, 1 M NaCl, 4 mM CaCl_2_, 1 mM PMSF, 0.01% BSA). The extractions were performed in conditions similar to binding with a 1-h incubation and the extracts were pooled together. Coniferyl alcohol (per)oxidase activities were measured from the supernatant after the binding (unbound activity) and the extraction steps (bound activity).

The polymers for binding assays were prepared as follows: extracellular lignin was harvested from the suspension culture by centrifugation, washed briefly with water and then 5 times with the extraction buffer (see above) to remove the native, lignin-bound proteins. After washing twice with water, lignin was lyophilized and ground to a fine powder in a mortar. This extracellular lignin sample was further deglycosylated by treating the polymer (250 mg suspended in 50 mM Na-acetate, pH 5.0) with a mixture of several glycosyl hydrolases (described in Virkki et al., 2008) at 37°C for 48 h. At 24 h, 5 mg of pectolyase (Sigma-Aldrich) was added to the reaction to increase the degradation of pectic polysaccharides. After hydrolysis, enzymes were inactivated by boiling for 5 min and the extracellular lignin was washed twice with water, twice with 1 M NaCl, and finally three times with water. The extracellular lignin control was treated similarly except that the enzymes were replaced with buffer only. DHP and MWL were washed, lyophilized and ground to a fine powder in a mortar similarly to the extracellular lignin sample.

### Determination of the carbohydrate component of extracellular lignin

Extracellular lignin was washed with water and then extracted with acetone to remove low-molecular-weight phenolic compounds, lyophilized and further dried in a vacuum oven at 40°C for 1 h. The sample (5 mg; in duplicates from three separate tissue cultures) was degraded by acid methanolysis essentially as described in Sundberg et al. (1996). Released monosaccharides were trimethylsilylated before gas chromatographic separation, which was performed on HP 6890N GC equipped with a flame ionization detector (Agilent Technologies) using a DB-1 (30 m, 0.25 mm i.d., 0.25 μm film thickness) capillary column from Agilent Technologies. Oven temperature profile was as follows: 150°C for 3 min; +1°C/min until 155°C; hold for 1 min; +2°C/min until 200°C; +20°C/min until 325°C. Arabinose, xylose, rhamnose, glucuronic acid, galacturonic acid, mannose, galactose and glucose were used as standards and sorbitol as an internal standard for quantification.

## Results

### Cloning of cDNA molecules encoding extracellular lignin-bound peroxidases

The Norway spruce tissue culture used in our study secretes proteins into the culture medium; part of these are bound to the extracellular lignin and can be extracted with a buffer containing 1 M NaCl. An isoelectric focusing gel stained for peroxidase activity showed that some of the extracted proteins with an isoelectric point (pI) ~10 had peroxidase activity (Koutaniemi, 2007). These peroxidases were partially purified, and amino acid sequences for several internal peptides were determined using LC-MS/MS tandem mass spectrometry (Supplementary Table 2). Based on the sequence data, cDNA molecules encoding the peptides were isolated. Three full length cDNAs were obtained, each coding for a few distinct amino acid sequences obtained during peptide sequencing (Supplementary Figure 1). The three cDNA sequences were designated as *PaPx16, PaPx17*, and *PaPx18* (EMBL: AM293546, AM293547, and AM293548, respectively). *PaPx16* and *PaPx17* are both translated into 341 amino acids. They are close homologs, the encoded amino acid sequence differing at eight positions. The *PaPx18* cDNA codes for a polypeptide of 310 amino acids. All three cDNAs encode a predicted signal sequence. The predicted pIs for mature PaPx16, PaPx17, and PaPx18 are 9.1, 9.2, and 8.4, respectively. The lower pI compared with the one determined from an isoelectric focusing gel is probably due to the bound Ca^2+^ ions and the heme, which increase the pI of the correctly folded peroxidase by up to 2 pI units (Welinder et al., 2002)

We searched the corresponding sequences from the Norway spruce genome (Nystedt et al., 2013). The closest homolog for *PaPx16* and *PaPx17* is MA_3486g0010, and for *PaPx18* MA_10432379g0020. Both of the genes have some expression in early wood (phloem+cambium+xylem; Nystedt et al., 2013).

### Peroxidase and laccase isoenzymes differ in polymer oxidation

Formation of radicals on lignin oligo- and polymers is a prerequisite for the polymer growth. Therefore, peroxidases that are involved in lignin polymerization should also be able to oxidize polymeric lignin. DHP produced from coniferyl alcohol with HRP was prepared as a near-authentic substrate, but we could not reliably apply the UV-Vis spectroscopic method of Sasaki et al. (2004) due to a limited amount of enzyme at hand. Instead, we used cytochrome *c* as a proxy high-molecular-weight substrate for peroxidases (Sasaki et al., 2004). We compared the ability of soluble culture medium proteins, extracted lignin-bound proteins and HRP to oxidize cytochrome *c*. Microperoxidase consisting of a heme with 11 amino acids attached was used as a positive control, and it was the most efficient of all tested enzymes in cytochrome *c* oxidation, reaching up to 35% of the oxidation rate for coniferyl alcohol (Table 2). Out of spruce native peroxidases, soluble culture medium peroxidases showed the highest oxidation rate, 1.8% of the rate for coniferyl alcohol. This was ca. 10-fold higher than that for extracted lignin-bound peroxidases, which was again 10-fold higher than that for HRP (Table 2).

**Table 2:** **Oxidation of cytochrome ***c*** by peroxidases (CytC prx) or dehydrogenation polymer (DHP) by laccases as a percentage of oxidation of coniferyl alcohol using soluble culture medium peroxidases (Pa-SL), lignin-bound peroxidases (Pa-BD), horseradish peroxidase (HRP), microperoxidase (MP), ***T. hirsuta*** laccase (Thi LAC) and ***M. albomyces*** laccase (Mal LAC); ***n*** = 3**.

**Protein sample**	**CytC prx oxidation (% of coniferyl alcohol oxidation)**	**DHP oxidation (% of coniferyl alcohol oxidation)**
Pa-SL	1.8 ± 0.2	
Pa-BD	0.19 ± 0.05	
HRP	0.018 ± 0.002	
MP	35.5 ± 1.5	
Thi LAC		64.9 ± 5.5
Mal LAC		13.8 ± 1.6

With laccases, assessment of DHP oxidation was possible by measuring oxygen consumption. Of the two laccases studied here, the rate of DHP oxidation for *T. hirsuta* laccase was ca. 5 times higher than for the *M. albomyces* laccase when compared with the rate of coniferyl alcohol oxidation (Table 2).

### Polymer-oxidizing enzymes produce natural-like lignin *in vitro*

To study the structural similarity of DHPs synthesized with various peroxidases and laccases, the distribution of the bond types in the resulting polymers were determined by quantitative 2D NMR. Standard scores were calculated for each result in order to give an equal weight to each bond type, and Pearson correlation coefficients were calculated to the bond distribution obtained for Norway spruce MWL (Table 3). The Pearson correlation coefficient provides a single measure, ranging from −1 to 1, that describes the similarity of the DHP to MWL.

**Table 3:** **Structural composition of natural and synthetic lignin samples determined using Q-HSQC and QQ-HSQC**.

**Sample**	**Activity**	**Pearson**	**Yield**	**β-*O*-4%**		**β-5%**		**β-β%**		**5-5-*O*-4%**	
**NORWAY SPRUCE LIGNIN**											
MWL^a^	–	+1.0	–	66.7 ± 1.0	*z* = +2.8	20.1 ± 0.4	*z* = −2.5	6.5 ± 0.1	*z* = −3.0	6.5 ± 0.1	*z* = +2.4
**NORWAY SPRUCE EXTRACELLULAR LIGNIN AND POLYMERS PRODUCED WITH ENZYMES ISOLATED FROM THE TISSUE CULTURE**											
EL^a^	–	+0.7	–	50.7 ± 0.6	*z* = +0.6	24.8 ± 0.2	*z* = −1.7	18.6 ± 0.0	*z* = +0.2	6.2 ± 0.2	*z* = +2.2
DHP/Pa-BD	200 nkat	+0.9	68 ± 5%	54.2	*z* = +1.1	26.8	*z* = −1.3	15.6	*z* = −0.6	3.4	*z* = +0.8
DHP/Pa-BD	5 nkat	−1.0	5 ± 2%	39.4 ± 0.3	*z* = −1.0	38.7 ± 0.6	*z* = +0.9	20.7 ± 0.3	*z* = +0.8	1.3 ± 0.0	*z* = −0.4
DHP/Pa-SL	200 nkat	−0.9	41 ± 6%	44.3 ± 0.0	*z* = −0.3	34.7 ± 0.2	*z* = +0.2	19.1 ± 0.4	*z* = +0.4	1.9 ± 0.1	*z* = 0.0
DHP/Pa-SL	5 nkat	−1.0	4 ± 2%	40.2 ± 1.2	*z* = −0.9	37.6 ± 1.3	*z* = +0.7	21.1 ± 0.3	*z* = +0.9	1.1 ± 0.2	*z* = −0.5
**POLYMERS PRODUCED WITH ENZYMES FROM NON-LIGNIN-FORMING SOURCES**											
DHP/HRP	20,000 nkat	–	58 ± 3%	Insoluble product							
DHP/HRP	5000 nkat	–	58 ± 4%	Insoluble product							
DHP/HRP^a^	1100 nkat	−0.9	41 ± 7%	45.4 ± 1.1	*z* = −0.2	35.4 ± 1.0	*z* = +0.3	18.2 ± 0.6	*z* = +0.1	1.0 ± 0.0	*z* = −0.5
DHP/HRP	200 nkat	−0.9	39 ± 3%	44.2 ± 0.7	*z* = −0.3	36.5 ± 1.1	*z* = +0.5	18.4 ± 0.1	*z* = +0.2	0.9 ± 0.0	*z* = −0.6
DHP/HRP	5 nkat	–	No recoverable product								
DHP/Mal	300 nkat	−1.0	28 ± 2	41.4 ± 0.2	*z* = −0.7	37.6 ± 0.3	*z* = +0.7	20.0 ± 0.1	*z* = +0.6	0.9 ± 0.0	*z* = −0.6
DHP/Thi	300 nkat	+0.7	48 ± 2	52.1 ± 0.9	*z* = +0.8	31.9 ± 0.4	*z* = −0.3	14.9 ± 0.4	*z* = −0.8	1.1 ± 0.0	*z* = −0.5
DHP/HRP+Thi	150 + 150 nkat	+0.6	39 ± 4	52.0 ± 0.8	*z* = +0.7	32.0 ± 0.0	*z* = −0.3	15.1 ± 0.1	*z* = −0.7	1.0 ± 0.0	*z* = −0.5
DHP/HRP+Mal	150 + 150 nkat	−1.0	29 ± 4	42.6 ± 0.2	*z* = −0.6	37.2 ± 0.3	*z* = +0.7	19.3 ± 0.2	*z* = +0.4	0.9 ± 0.0	*z* = −0.6

*Activity column indicates the total coniferyl alcohol (per)oxidase activity used in the production of DHPs. Pearson column gives a Pearson correlation of the standard scores of a sample with milled wood lignin (MWL). Yield column gives the yields of DHP synthesis as a percentage of the maximum theoretical yield. The final four columns give the proportions of the four main lignin substructures (β-O-4, β-5, β-β and 5-5-O-4) as percentages of the total 2D NMR signal from these structures and standard scores (z) calculated from these values. EL, extracellular lignin; DHP, dehydrogenation polymer; Pa-BD, total proteins extracted from extracellular lignin; Pa-SL, total soluble proteins from the culture medium; HRP, horseradish peroxidase; Mal, Melanocarpus albomyces laccase; Thi, Trametes hirsuta laccase*.

a *Results published previously in Koutaniemi et al. (2005)*.

As lignin is a heterogeneous polymer that is composed of different structural units, its structure can be described using the percentage values of different monomer unit linkages (Table 3). MWL contained a high proportion of β-*O*-4 (66.7%) and of 5-5-*O*-4 substructures (6.5%), corresponding well with earlier results (Heikkinen et al., 2003). Extracellular lignin had an equally high proportion of 5-5-*O*-4 while the content of β-*O*-4 was slightly lower. The DHP produced with 200 nkat (measured as coniferyl alcohol peroxidase activity) of enzymes extracted from the extracellular lignin resembled MWL more than any other DHP, and gave the highest yield of the polymer (Table 3). Very high activity (5000 and 20,000 nkat) of HRP produced good yields of the polymer, but these DHPs were resistant to dissolution for NMR, possibly indicating crosslinking reactions taking place. The soluble culture medium enzymes (200 nkat) produced DHPs that were structurally different from the MWL samples, containing less β-*O*-5 and 5-5-*O*-4 linkages (Table 3). A similar result was obtained with HRP (200 nkat) and lower activities (5 nkat) of the extracellular lignin-extracted enzymes. The second best structural correlation with MWL after the DHP produced with the extracellular lignin-extracted enzymes (at 200 nkat) was observed in DHP produced using *T. hirsuta* laccase (Table 3) with the ability to oxidize lignin polymers efficiently (Table 2). Interestingly, the DHP produced with *T. hirsuta* laccase was more similar to MWL than that produced with *M. albomyces* laccase, the enzyme with a lower polymer-oxidizing activity. DHPs produced with mixtures of equal amounts of each laccase and HRP (150 nkat each) showed that the *T. hirsuta* laccase dominated over HRP the structure of the resulting polymer. With HRP and *M. albomyces* laccase the polymer was similar to those produced with either enzyme alone.

### Extracellular lignin contains pectic polysaccharides

NMR analyses of extracellular lignin have shown the presence of carbohydrates (I. Kilpeläinen, unpublished data). These lignin-associated carbohydrates remained bound to lignin after washing with buffered salt solution (1 M NaCl), suggesting the presence of covalent linkages between the sugar units and lignin. The carbohydrate component of the extracellular lignin was studied in more detail using gas chromatography after degradation of the carbohydrate component by acid methanolysis. The total carbohydrate content in the extracellular lignin was 166 ± 27 μg/mg dry weight (Table 4). Galacturonic acid (GalA), arabinose (Ara) and galactose (Gal) were detected in larger amounts, while rhamnose (Rha), xylose (Xyl), glucose (Glc) and mannose (Man) were present at lower levels. The abundance of GalA suggests the presence of pectic polysaccharides, mainly homogalacturonan (Albersheim et al., 2011; Atmodjo et al., 2013). However, the presence of Rha, Ara and Gal also point to the presence of rhamnogalacturonan I, which contains a backbone of repeating α-GalA-(1-2)-α-l-Rha-(1-4) disaccharide, which is substituted with side chains containing mostly Ara and Gal (Atmodjo et al., 2013). The detected Man and Glc suggest the presence of (galacto)glucomannan, which is the main hemicellulosic polysaccharide in softwoods (Rowell et al., 2005).

**Table 4:** **Sugar composition of the carbohydrate fraction of the extracellular lignin, determined after acid methanolysis and trimethylsilylation using gas chromatography (average ± standard error of the mean, ***n*** = 5)**.

**Monosaccharide**	**μg (mg dry weight)^−1^**
Galacturonic acid	60.1 ± 7.6
Arabinose	57.4 ± 7.8
Galactose	32.3 ± 5.2
Glucose	5.3 ± 1.6
Rhamnose	3.9 ± 0.6
Mannose	2.1 ± 0.8
Xylose	1.7 ± 0.4
Fucose	1.5 ± 0.3
Glucuronic acid	0.9 ± 0.5

### Extracellular lignin-extracted peroxidases bind to lignin-like polymers

To determine which component of the extracellular lignin the peroxidases are bound to, we studied the ability of the total soluble culture medium proteins and the total proteins extracted from the extracellular lignin to bind different phenolic and carbohydrate polymers. The polymers included extracellular lignin before and after enzymatic degradation of the carbohydrate component, DHP produced from coniferyl alcohol with HRP, MWL and two anionic carbohydrate polymers, calcium polygalacturonate and calcium alginate.

**Figure 2:**
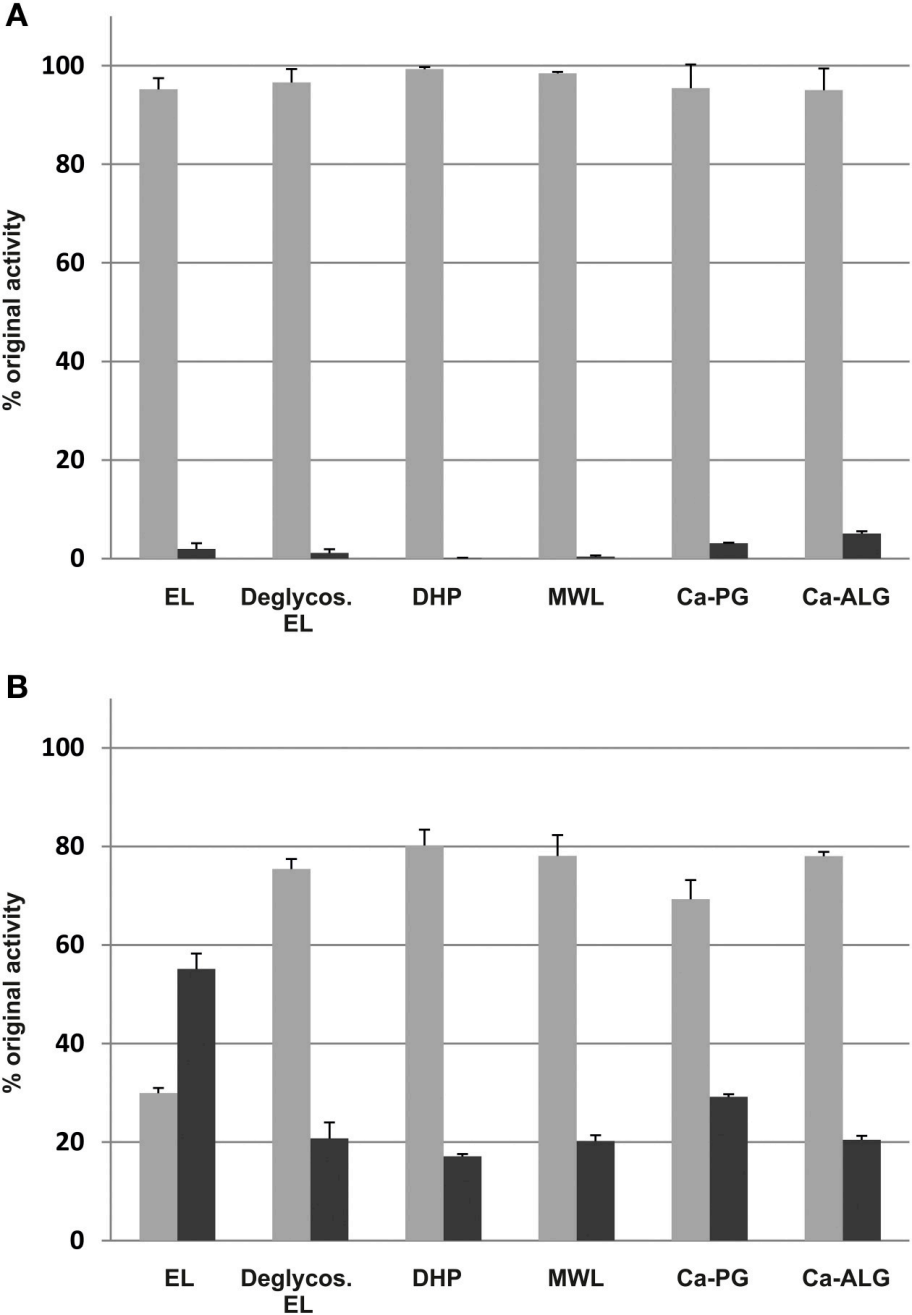
**Binding of the soluble culture medium proteins (A), and the extracted, lignin-bound proteins (B) to lignin-like and anionic polysaccharide polymers**. EL, extracellular lignin; Deglycos. EL, extracellular lignin after the glycosyl hydrolase treatment; DHP, dehydrogenation polymer from coniferyl alcohol made with HRP; MWL, milled wood lignin; Ca-PG, Ca-polygalacturonate; Ca-ALG, Ca-alginate. Light gray, unbound fraction; dark gray, bound fraction.

**Figure 3:**
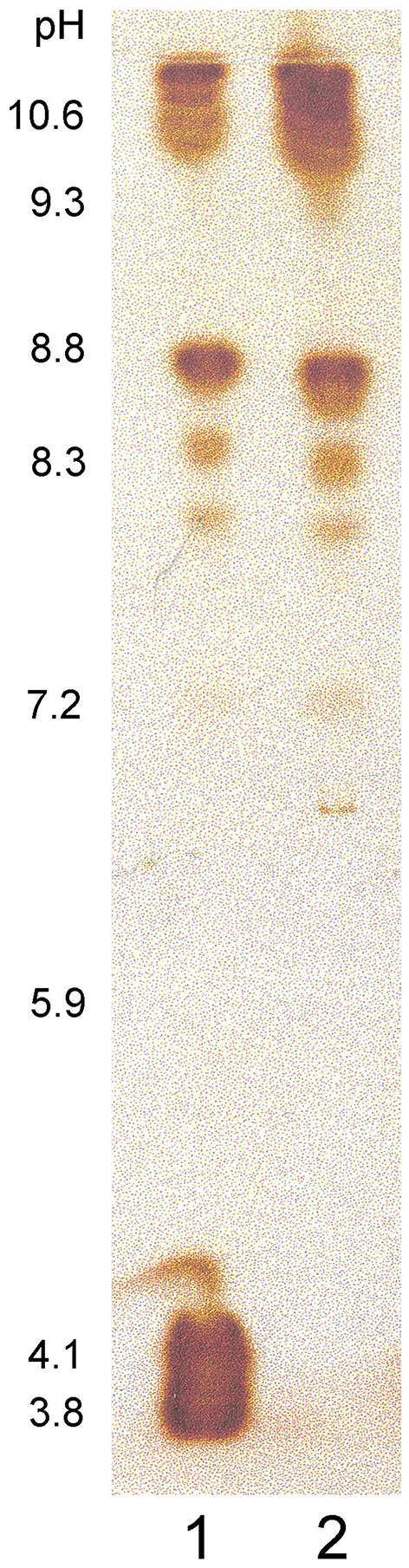
**Norway spruce culture medium proteins separated in an isoelectric focusing gel and stained for peroxidase activity**. (1) Proteins unbound to MWL, (2) Proteins bound to MWL. Numbers beside the IEF gel indicate the determined pH gradient in the gel.

Cationic peroxidases extracted from the extracellular lignin had a significant affinity to all lignin polymers tested (Figure 2). The binding was greatest to extracellular lignin itself, with 55 ± 3% of the activity bound, but significant binding was also observed to all other polymers tested, with binding between 17 and 29%. Enzymatic degradation of the extracellular lignin-bound carbohydrates reduced, but did not eliminate this binding (Figure 2). In contrast, the binding of the soluble culture medium peroxidases to the polymers was limited. The highest apparent affinity was to calcium alginate, which bound 5 ± 0.5% of the soluble peroxidase activity (Figure 2). Only basic peroxidases from the culture medium bound to lignin as shown in the IEF gel stained for peroxidase activity (Figure 3).

The ability of *T. hirsuta* laccase, *M. albomyces* laccase and HRP to bind to the DHP produced by HRP was also measured. Under the experimental conditions 8.0 ± 1.1% of the *T. hirsuta* laccase activity and 3.8 ± 3.1% of that of *M. albomyces* were bound to the polymer. No significant binding of HPR could be detected (data not shown).

## Discussion

In this work we have investigated the role of oxidative enzymes, isolated from Norway spruce, on lignin structure with the help of DHPs produced from coniferyl alcohol. The results show that different enzymes, or varying enzyme activity amounts under otherwise identical conditions, produce phenolic polymers with different proportions of the lignin substructures.

Lignin polymer grows when an unpaired electron in the polymer radical forms a covalent bond with an unpaired electron in the monolignol radical. Thus, all enzymes that can produce a polymer from monolignols must also oxidize the polymeric substrate (directly or indirectly). However, only high oxidation rate of the polymer will produce intramolecular cross-links, such as the 5-5 bond that occurs in the 5-5-*O*-4 substructure typical for conifer wood (Karhunen et al., 1995; Kukkola et al., 2003). This suggests that the failure of most *in vitro*-polymers to mimic natural lignins results from an insufficient oxidation of the growing polymer.

The present study shows that the Norway spruce cell suspension culture has a subset of peroxidases, which can bind to lignin-like and pectic polymers (Figures 2, 3), and which can oxidize polymeric lignin (Table 3). This set of extracellular lignin-extracted peroxidases, as well as *T. hirsuta* laccase that also has a considerable polymer-oxidizing ability (Table 2), produced DHPs in a high yield (>50%) and with a high proportion of β-*O*-4 substructures (Table 3). This is considered as a signature for end-wise polymerization (Sarkanen, 1971), requiring oxidation of the polymer to proceed. Enzymes such as HRP or *M. albomyces* laccase with lower polymer-oxidizing ability (Table 2), when dosed at the same level, produced a condensed polymer with less β-*O*-4 and a high proportion of β-5 linkages (Table 3). Interestingly, DHP produced with the extracellular lignin-extracted enzymes (at 200 nkat) was close to natural spruce lignin (MWL), more similar than any of the DHPs produced with other enzymes tested. The main contributor to this similarity was, in addition to the β-*O*-4 content, the relatively high content of 5-5-*O*-4 bonds (Table 3). With a more sparing amount of the lignin-extracted enzymes (5 nkat) we obtained a low polymer yield and a bond distribution similar to that in DHP produced with HRP at a 200-nkat dosage (Table 3).

This observation led to a suggestion that the key factor in the synthesis of natural-like lignin is not the polymer-oxidizing ability *per se*, but instead, the ratio of polymer to monomer oxidation occurring in the actual reaction. One way to increase polymer oxidation is to have enzymes that are specialized in this function; however, also an increase in the enzyme activity should have the same effect since it leads to a rapid consumption of the monomers. Therefore, also HRP should be able to produce a natural-like lignin if sufficient amount of the activity was applied. Indeed, experiments with 5000 nkat and 20,000 nkat of HRP did produce a precipitate in a high yield (58%, Table 3). Contrary to most of the DHPs assayed, but similar to some repetitions of the experiment with 200 nkat of the extracellular lignin-extracted enzymes, these polymers could not be made soluble for the NMR analysis of the actual bond distributions, which is a possible indicator of crosslinking of the polymer.

Increasing the oxidative enzymatic activity to produce more natural-like lignins is essentially the same but an extended effect, which is known from comparisons of “*zutropf*” and “*zulauf*” polymers, where “*zutropf*” polymers have a higher molecular mass and a structure that is more similar to that in natural lignins (Freudenberg, 1956). *Zutropf* refers to gradual feeding of the substrates into the reaction vessel, i.e., increasing the enzyme to substrate ratio (somewhat simulating lignin biosynthesis), while in a *zulauf* reaction all substrates are added in the beginning of the reaction. Syrjänen and Brunow (2000) utilized the same principle in an elegant experimental setup, where coniferyl alcohol was provided very slowly to a HRP-containing solution through a dialysis membrane, producing a native-like polymer with increased proportion of 5-5-*O*-4 bonds. Méchin et al. (2007) have also tested how peroxidase activity affects the DHP structure, concluding however that for *zutropf* polymers the structure was more natural-like when lower enzyme activities were used. This seemingly contradictory result is at least partly due to a different measure of “natural-like”, which for Méchin et al. (2007) was the abundance of β-*O*-4 bonds. In the present study, we used ratios of all four bond types, with 5-5-*O*-4 having a high weight. As stated above, this bond type forms only when the growing polymer is oxidized efficiently. In fact, high redox potential of the guaiacyl groups prevents their cross-coupling until the concentration of the more reactive coniferyl alcohol monomers is sufficiently low and approaches zero (Brunow et al., 1998). In xylem cells the concentration of monolignols approaches zero after programmed cell death of lignifying cells. Indeed, in spruce this is accompanied by an increase in 5-5-*O*-4 structures in the S3 layer of the secondary cell wall (Kukkola et al., 2003).

Our results are in line with the view that lignin polymerization is a stochastic process where the probabilities of various bond types are determined by the concentration ratios, resonance structures and oxidation potentials of the radicals involved (Adler, 1977; Grabber et al., 1996; Syrjänen and Brunow, 1998, 2000; Sasaki et al., 2004, 2008). As the latter two are essentially constant, the concentration ratio of polymer to monomer radicals emerges as a principal way how lignin structure is determined. Oxidative enzymes differ in their ability to accept large substrates. In poplar, one of the secreted, cell wall-bound peroxidases showed increased polymer-oxidizing activity that related to tyrosine residues that relay electrons to the surface of the enzyme (Sasaki et al., 2008; Shigeto et al., 2012), a similar solution as in fungal enzymes that oxidatively degrade lignin (Ruiz-Dueñas and Martínez, 2009). In the spruce tissue culture, those peroxidases that produced the natural-lignin-like polymer also efficiently bound to the substrate, i.e., to extracellular lignin (Figure 2). This binding may be partly *via* electrostatic interactions to negatively charged pectic polysaccharides that are attached to extracellular lignin (Table 4). As the glycosyl hydrolase treatment only partially suppressed the re-binding of the enzymes to deglycosylated extracellular lignin (Figure 2), some direct interaction with lignin seems likely. This is supported by the fact that peroxidases extracted from the extracellular lignin bound also to DHP produced with HRP in the absence of carbohydrates. Interestingly, DHPs produced in the presence of pectin or xylan form covalently bound lignin-carbohydrate complexes, increasing the molar mass and solubility of the DHPs, as well as the content of β-*O*-4 structures using *zutropf* feeding of the reactants (Cathala and Monties, 2001; Barakat et al., 2007; Li et al., 2015). This emphasizes the role of the polysaccharide matrix on the outcome of lignin polymerization also *in vivo*.

It is too early to say if the results of Sasaki et al. (2008) and the experiments described here represent angiosperm and gymnosperm solutions for lignin polymerization, but they suggest that among plant peroxidases, some have evolved and specialized in the task of lignin polymerization. According to the emerging insight, plants have five principal ways to control lignin structure: The ratio of different monolignols synthesized, the rate of transport of monolignols into the apoplast, pH of the apoplast and the quantity and the properties of the oxidative enzymes. These simple parameters may be sufficient to control the variation in lignin that can be observed in both gymnosperm and angiosperm wood.

## Author contributions

TT, IK, LS, SK, AK, and TW conceived and designed the experiments. TW, SK, AK, IS, MT, IK, and TT performed the research. TW, SK, AK, and TT wrote the manuscript. All authors read and approved the manuscript.

### Conflict of interest statement

The authors declare that the research was conducted in the absence of any commercial or financial relationships that could be construed as a potential conflict of interest.

## Abbreviations

DHP: dehydrogenation polymer
HRP: horseradish peroxidase
pI: isoelectric point
PMSF: phenylmethylsulfonyl fluoride.
