# Supplementary material for: Peroxidases Bound to the Growing Lignin Polymer Produce Natural Like Extracellular Lignin in a Cell Culture of Norway Spruce
Source: Front Plant Sci. 2016 Oct 18;7:1523. doi: 10.3389/fpls.2016.01523 (PMC5067304; doi:10.3389/fpls.2016.01523)
Supplement: Supplementary file 1 [file Table1.PDF]

Supplementary Material

Peroxidases bound to the growing lignin polymer produce natural like extracellular lignin in a cell culture of Norway spruce

Tino Warinowski, Sanna Koutaniemi, Anna Kärkönen, Ilari Sundberg, Merja Toikka, Liisa Kaarina Simola, Ilkka Kilpeläinen and Teemu Heikki Teeri*

*** Correspondence:** Corresponding Author: teemu.teeri@helsinki.fi

Supplementary Table 1. Primers used to clone three full-length cDNAs of Norway spruce peroxidases putatively bound to extracellular lignin.

	Primer pair sequences	Product
**Pr6**<br>**Pr12**	CTGGTRCTGTAGGAYTCAAC<br>GGWGTCGTGTCRTGTGCWGACATTCTC	Partial PaPx16 and,<br>partial PaPx17
**Pr1B**<br>**Pr14**	CTCATCTCAMTITTTCARGCTCA<br>CTTCCTGCAGTTTTTGCGRATTTG	Partial PaPx18
**Pr15**<br>**UPM**	GTTTGAGGACACACGGCCCTCAGATGC	5’ UTR of PaPx16 and<br>5’ UTR of PaPx17
**Pr17**<br>**UPM**	ATGGGCTCTAAAATTCACGCACCGCGC	5’ UTR of PaPx18
**Pr19**<br>**UPM**	GTTCAGACATCGCATCGGTGCTCCC	Full-length PaPx17
**Pr20**<br>**UPM**	CGCGGGGGGCAAGATTGAAACTCTTAGC	Full-length PaPx16
**Pr23**<br>**UPM**	CGCGGGGGGCAAGATTGAAACTCTTAGC	Full-length PaPx18

UPM = Universal Primer A Mix from the SMART RACE cDNA Amplification Kit (BD Biosciences Clontech)
