# Supplementary material for: Peroxidases Bound to the Growing Lignin Polymer Produce Natural Like Extracellular Lignin in a Cell Culture of Norway Spruce
Source: Front Plant Sci. 2016 Oct 18;7:1523. doi: 10.3389/fpls.2016.01523 (PMC5067304; doi:10.3389/fpls.2016.01523)
Supplement: Supplementary file 2 [file Table2.PDF]

Supplementary Material

Peroxidases bound to the growing lignin polymer produce natural like extracellular lignin in a cell culture of Norway spruce

Tino Warinowski, Sanna Koutaniemi, Anna Kärkönen, Ilari Sundberg, Merja Toikka, Liisa Kaarina Simola, Ilkka Kilpeläinen and Teemu Heikki Teeri*

*** Correspondence:** Corresponding Author: teemu.teeri@helsinki.fi

Supplementary Table 2. Peptides obtained by mass spectrometric analysis of tryptic digests of partially purified peroxidases extracted from the extracellular lignin.

Identical matches to the cloned peroxidase sequences	Similarity to the cloned sequences or other spruce peroxidase sequences
PGVVSCAD(I/L)(I/L)<br>SGVVSCAD(I/L)(I/L)A(I/L)S<br>(I/L)(L/I)S(I/L)FQAQ<br>VESYSTSTHAFFK<br>DV(I/L)DT(I/L)K	(I/L)SP(L/I)TGTgs(l/i)q<br>cadGS(I/L)(L/I)(L/I)DDTC<br>SGVVSCAD(I/L)(L/I)S<br>VT(L/I)GGPTWTVM<br>NEFLDFAAA<br>(I/L)FP(L/I)TGSQG(I/L)QR<br>NSSSFYDK<br>(I/L)(I/L)SFSSASS<br>D(L/I)VA(L/I)SGAHT(L/I)GQSR<br>D(L/I)VA(L/I)Sqht(l/i)ma<br>VD(L/I)VA(L/I)Sqhya

Isoleucine (I) and leucine (L) cannot be discriminated with the method used, hence, both alternatives are shown.
