# Supplementary material for: Peroxidases Bound to the Growing Lignin Polymer Produce Natural Like Extracellular Lignin in a Cell Culture of Norway Spruce
Source: Front Plant Sci. 2016 Oct 18;7:1523. doi: 10.3389/fpls.2016.01523 (PMC5067304; doi:10.3389/fpls.2016.01523)
Supplement: Supplementary file 3 [file Image1.pdf]

Supplementary Material

Peroxidases bound to the growing lignin polymer produce natural like extracellular lignin in a cell culture of Norway spruce

Tino Warinowski, Sanna Koutaniemi, Anna Kärkönen, Ilari Sundberg, Merja Toikka, Liisa Kaarina Simola, Ilkka Kilpeläinen and Teemu Heikki Teeri*

*** Correspondence:** Corresponding Author: teemu.teeri@helsinki.fi

```
PaPx16     1 MAQIKHSPGLTLQFLSVLITGLALMLWIQTVDAQSCNGLSHHFYYKSCPKAQAIIKSVVE  60
PaPx17     1 MAQIKHSPGLTLQFLSVLITGLALMLWIQTVDAQSCNGLSHHFYYKSCPKAQAIIKSVVE  60
PaPx18     1 ----------------------MVFIVCS-ITHTANGQLSSTFYAQSCPTALSVVKAAVR  37
CpAPX      1 ---------------------MALLFFLALLFGASYAQLTETFYDQTCPRLPNIVRQEVK  39
PaPx4      1 -------------MKTLACIVLCVLCISS-INNAAHGQLTSTFYNKLCPTALSIVKAAVN  46
PaPx5      1 -----------MMMRTLVCIGVMAVLLCSININAVNGQLSSTFYAKSCPRVQSIVKTVVK  49
                                     .             *:  ** : **    :::  *.

PaPx16    61 DAVRKEAGMAASLLRLHFHDCFVKGCDGSILLDDTSSFTREKTANPNRNSVRGFGVVDQI 120
PaPx17    61 DAVRKEARMAASLLRLHFHDCFVKGCDGSILLDDTSSFTGEKTANPNRNSVRGFGVVDQI 120
PaPx18    38 QAVANEKRMGASLLRLHFHDCFVNGCDGSVLLDDSSTITGEKTANPNANSARGFDVIDTI  97
CpAPX     40 RAIETDIRAGAKLIRFHFHDCFVQGCDGSVLLEDAPGIDSELNGLG-NLGIQGLEIVDAI  98
PaPx4     47 KAVNNEKRMGASLLRLHFHDCFVNGCDGSILLDDNSTFTGEKTALPNANSVRGFDVIDTI 106
PaPx5     50 QAVAKEKRMGASLVRLHFHDCFVNGCDGSILLDDNATFTGEKTAGPNANSARGFDVIDTI 109
              *: .:   .*.*:*:*******:*****:**:*   :  * ..     . :*: ::* *

PaPx16   121 KCELEKACPGVVSCADILAVAARDSVGFSGGPFWKVLLGRRDSRSASKSGANNDIPGPNS 180
PaPx17   121 KSELEKACPGVVSCADILAVAARDSVGFSGGPFWKVLLGRRDSRSASKSGANNDIPGPNS 180
PaPx18    98 KSNVEKACSGVVSCADILAISARDSVVELGGPSWTVMLGRRDSTTASKNGANNNIPPPTS 157
CpAPX     99 KAAVESECPGVVSCADVLALAAKQSVDVQGGPSWRVLFGRRDSRTANRTGA-DELPSPFE 157
PaPx4    107 KTQVEAACSGVVSCADILAIVARDSVVQLGGPTWTVLLGRRDSTTASLSAANNNIPSPAS 166
PaPx5    110 KTQVEAACSGVVSCADILTIAARDSIVELQGPTWTVMLGRRDSPTASLSAANNNIPSPAS 169
             *  :*  * *******:*:: *::*:    ** * *::***** :*. ..* :::* * .

PaPx16   181 THQTLETKFKRQGLNVVDLVALSGAHTIGLARCSSFKARLYNQTVNGKPDPTLDTTYLKH 240
PaPx17   181 THQTLETKFKRQGLNVVDLVALSGAHTIGLARCSSFKARLYNQTVNGKSDPTLDTTYLKH 240
PaPx18   158 SLSNLISLFQAQGLSTKEMVALSGGHTIGQARCVNFRAHIYNET-------NIDSTYSTS 210
CpAPX    158 TLEPLKQKFEALGLDSTDLVAPSGAHTFGRSRCMFFSGRFSNFNGTGQPDPALDPAYRQE 217
PaPx4    167 NLSALISSFTAHGLSTKDLVALSGGHTIGQARCTTFRARIYNES-------NIDTSFATS 219
PaPx5    170 SLSTLITSFQNHGLSTKDLVALSGAHTIGQSRCAFFRTRIYNES-------NINAAFATS 222
             . . *   *   **.  ::** **.**:* :**  *  :: * .        :: ::

PaPx16   241 LRAVCPQTG-TDDNQTTPLDPVTPIRFDINYYDNVVAGKGLLASDEILYSTKGSRTVGLV 299
PaPx17   241 LRAVCPQTG-TDDNQTTPLDPVTPIKFDIDYYDNVVAGKGLLASDQILYSTKGSRTVGLV 299
PaPx18   211 LQSKCPSTAGSGDSNLSPLDYVTPTAFDKNYYSNLKSKKGLLHSDQELFNG--GSTDSQV 268
CpAPX    218 LERACTDG-----ETRVNFDPTTPDTFDKNYYTNLQANRGLLTSDQVLFSTPGADTIEIV 272
PaPx4    220 VKSSCPSAG--GDNTLSPLDLATPTTFDNKYYTDLGNRKGLLHSDQQLFSG--GSTNSQV 275
PaPx5    223 VKPNCPSAG--GDNTLSPLDVVTPTTFDNKYYSNLKVQKGLLHSDQQLFNG--GSTDSQV 278
             :.  * .      .    :* .**  ** .** ::   :*** **: *:.   . *   *
```

```
PaPx16  300 ESYSTSTHAFFKQFAASMIKMGNINPLTGSHGEIRKNCRRMN------------      341
PaPx17  300 ESYSTSMHAFFKQFAASMIKMGNINPLTGSHGEIRKNCRRMN------------      341
PaPx18  269 TTYASNQNSFFSDFAAAMVKMGNIKPLTGTSGQIRKNCRKPN------------      310
CpAPX   273 NRLGSREGTFFRQFRVSMIKMGNIRPLTPNQGEIRRNCRGVNELGGEAGHDVM      325
PaPx4   276 TTYSANQNTFFTDFAAAMVKMGNISPLTGTSGQIRKNCRKAN------------      317
PaPx5   279 TTYSTNQNSFFTDFAAAMVKMGNISPLTGTSGQIRKNCRKAN------------      320
               .:   :** :* .:*:***** *** . *:**:***  *
```

Supplementary Figure 1. Sequence alignment of the cloned peroxidase sequences PapPx16 (AM293546.1), PaPx17 (AM293547.1) and PaPx18 (AM293548.1) with spruce peroxidases PaPx4 (AJ809339.1) and PaPx5 (AJ809340.1), and the anionic pectin-binding peroxidase CpAPX (Y17192.1) from zucchini. In the latter, the pectin-binding arginine residues are shown in bold. The sequenced peptides are indicated in colour.
